# Authors’ Reply: Enhancing the Clinical Relevance of Al Research for Medication Decision-Making

**DOI:** 10.2196/72007

**Published:** 2025-02-18

**Authors:** Sarah E Vordenberg, Julianna Nichols, Vincent D Marshall, Kristie Rebecca Weir, Michael P Dorsch

**Affiliations:** 1 College of Pharmacy University of Michigan Ann Arbor, MI United States; 2 Sydney School of Public Health Faculty of Medicine and Health University of Sydney Sydney Australia; 3 Institute of Primary Health Care (BIHAM) University of Bern Bern Switzerland

**Keywords:** older adults, artificial intelligence, vignette, pharmacology, medication, decision-making, aging, attitude, perception, perspective, electronic heath record

We appreciate the insights regarding our manuscript, “Investigating Older Adults’ Perceptions of AI Tools for Medication Decisions: Vignette-Based Experimental Survey” [1], in the letter to the editor shared by Wang and Chen [2]. The letter emphasizes three key points: (1) older adults may encounter practical challenges with artificial intelligence tools, necessitating user testing and scenario simulations to enhance usability; (2) although our study considered demographic differences, it did not explore underlying cultural and socioeconomic factors, which calls for further research; (3) the complexity of real-world medication decision-making remains significant.

We acknowledge the limitations the correspondents highlight, particularly usability challenges for older adults and the need to examine underlying cultural and socioeconomic factors. Our vignette-based experiment served as an intentionally focused first step in understanding older adults’ interest in artificial intelligence–assisted medication decision support.

Wang and Chen [2] note that our previous work showed that medication decision-making is complex [3]. Indeed, various factors influence how patients make these decisions, including their attitudes, beliefs, and preferences regarding medications; the potential benefits and harms of the treatment under consideration; and contextual factors specific to the patient [4-6]. Our study’s experimental approach using vignettes, as indicated in the title, was selected to begin exploring this complex area in a controlled manner.

We agree that this initial study lays the foundation for future research that can tackle these important considerations through enhanced scenarios, user testing, and a more in-depth examination of sociocultural factors.
